# Psychopathological and Organic Features of Atypical Anorexia Nervosa in Developmental Age: A Systematic Review

**DOI:** 10.3390/pediatric16030049

**Published:** 2024-07-16

**Authors:** Jacopo Pruccoli, Francesca Chiavarino, Beatrice Valeriani, Maria Letizia Petio, Antonia Parmeggiani

**Affiliations:** 1 IRCCS Istituto delle Scienze Neurologiche di Bologna, Regional Center for Feeding and Eating Disorders in the Developmental Age, Child Neurology and Psychiatry Unit, 40138 Bologna, Italy; 2 Department of Medical and Surgical Sciences (DIMEC), University of Bologna, 40126 Bologna, Italy; 3 IRCCS Azienda Ospedaliero-Universitaria di Bologna, Clinical Nutrition and Metabolism Unit, 40138 Bologna, Italy

**Keywords:** atypical anorexia nervosa, developmental age, BMI, feeding and eating disorders, weight loss, neurobiology

## Abstract

Purpose: This study aimed to comprehensively report the epidemiological and clinical features of atypical anorexia nervosa (AAN) in children and adolescents. Methods: In May 2024, a systematic review was performed using Medline, Cochrane Library, ClinicalTrials.gov, and relevant websites. Following PRISMA guidelines, 234 articles were screened for studies on DSM-5-defined AAN. A standardized checklist—the JBI critical appraisal tool—was adopted in assessing methodology, and 13 retained studies passed the screening and critical appraisal process for the final review. The Newcastle–Ottawa Scale was utilized to assess the risk of bias in cohort and case–control studies, ensuring a comprehensive evaluation of methodological quality. Results: AAN prevalence in young age groups is 2.8%, with a cumulative 2.8% incidence over 8 years. Incidence is 366 per 100,000 person-years, and the average episode duration is 11.6 months, with a 71% remission rate. Diagnostic persistence for AAN is less stable than other restrictive feeding and eating disorders (FEDs). AAN individuals exhibit higher EDE-Q scores, more severe distress, and distinct BMI differences compared to those with anorexia nervosa and controls. The diagnostic transition from the DSM-IV to the DSM-5 shows that AAN patients are predominantly female, slightly older, and with higher weight. Conclusions: This study yields concrete insights into the features of AAN in the developmental age, highlighting demographic variations, clinical presentations, and treatment outcomes. Recognizing the unique challenges faced by AAN individuals is vital for tailoring effective interventions and improving overall care within the FED spectrum.

## 1. Introduction

### 1.1. Definition

Atypical anorexia nervosa (AAN) is a feeding and eating disorder (FED) marked by cognitive features such as fear of gaining weight and body image disturbance, as well as behavioral patterns like caloric restriction, purging, and binge eating. These characteristics are comparable to those observed in the more commonly recognized anorexia nervosa (AN), although individuals with AAN maintain a weight within the normal or higher range [1]. Studies on the topic agree that AAN is as severe as, if not more severe than, AN [2,3,4]. An increasing number of studies have recently investigated the main medical and psychopathological complications that accompany this condition, which currently represents an emerging topic in the scientific literature [5]. However, no comprehensive review exists collecting this information from a diagnostic and management perspective in developmental age and in the context of the most recent guidelines regarding the management of FEDs in individuals with higher weight (a condition that has been reported as premorbid, with respect to AAN [2]), including patients with AAN [6]. The present article aims to report on the existing evidence on the psychopathological and organic of AAN in children and adolescents in the medical, psychological, and nutritional fields.

### 1.2. Evolution of the Concept of “Atypical Anorexia Nervosa”

The concept of AAN refers to a clinical condition in mental health that is defined by a partial overlap with the diagnostic criteria of classic (“typical”) AN. Affected individuals, however, are within or above the normal weight range, despite significant weight loss [1]. The development of AAN as a diagnosis, first recognized and categorized with the publication of the fifth edition of the *Diagnostic and Statistical Manual of Mental Disorders* (DSM-5), has followed a complex process of scientific and clinical validation, in the absence, to date, of an unambiguous and simple identification [6,7].

The DSM-IV TR [8] documents the initial consideration of particular clinical conditions not overlapping with other clinical pictures: it is covered by creating the residual category “Eating Disorders Not Otherwise Specified” (EDNOS), which included, among others, individuals who met all criteria for AN, but whose weight was within the normal range [6]. The definition of specific criteria for AAN originally stems from the need to expand the diagnostic capacity of AN criteria [9]. The DSM-5 describes the simultaneous presence of these criteria as follows.

The advancement of research in the field of AN has led some researchers to theorize and identify more complex, nuanced, and less stereotypical clinical pictures. Recent research on AN encompasses themes on the vulnerability of the therapist, therapeutic bias, communication between clinicians and patients, and patients’ involvement in treatment [10]. It was eventually pointed out that the clinical (i.e., medical and psychopathological) impact of malnutrition can also be significant in normal-weight or overweight individuals [9]. Such evidence drew the attention of clinicians and researchers to psychopathological manifestations compatible with AN (e.g., phobia of gaining weight, body image disorder) that are present in individuals who do not meet the “underweight” criterion [2].

The DSM-5 further acknowledged and more accurately framed this condition by presenting the diagnostic group “Nutrition or Eating Disorders with Other Specification” (OSFED), which includes the five diagnostic constructs of atypical anorexia nervosa, bulimia nervosa (low frequency and/or limited duration), uncontrolled eating disorder (low frequency and/or limited duration), elimination conduct disorder, and night eating syndrome within the chapter “Feeding and Eating Disorders” [1].

### 1.3. AAN in the Recent Literature

The following section describes the specific diagnostic features of AAN while also reporting the main problems that researchers encountered in providing an unambiguous and clinically valid definition.

The diagnosis of AAN has been recently introduced in the “Feeding and Eating Disorders” section of the DSM-5. Only a few lines of the manual are devoted to the discussion of the diagnostic criteria for AAN, which prompted the following observations according to other literature opinions.

“All criteria for anorexia nervosa are met, except…”: by definition, the DSM-5 makes no distinction regarding the psychopathology of the two conditions (AAN and AN) (fear of weight gain and body image disturbance).

“… despite significant weight loss…”: clinicians and researchers have provided different interpretations of this description. The greatest challenge is to define the threshold for which weight loss can be considered “significant” in the absence of a specific indicator in the text, particularly in developmental age. Examples of operational definitions have included weight loss thresholds of 5%, 10%, 15%, 18–20%, and 25%, loss of 1.3 BMI points, and no significant weight loss [11,12,13,14]. A study by Forney et al. has specifically investigated this issue by testing the impact of three distinct criteria for defining “weight loss” (5%, 10%, and 15%) on prevalence and levels of clinical impairment in a group of individuals with AAN. Although prevalence was directly affected by the different criteria (with a 5% threshold, 13% of the women included in the study could have been diagnosed with AAN, but the percentage of women decreased to 2.8% when a 15% threshold was applied), the clinical profiles of the different groups thus obtained were similar. Such evidence has led the authors to emphasize the usefulness and homogeneity of the clinical construct of AAN, regardless of the weight thresholds identified [14]. It is also worth noting that the DSM-5 itself, in the section on AN, specifies that in the case of children and adolescents, clinicians should attend to instances of failure to achieve expected weight gains or to maintain a normal developmental trajectory (e.g., growth in height), not only to frank detections of weight loss [1].

“… the individual’s weight is within or above normal reference intervals”: the definition of standard reference intervals is a frequently addressed issue in AN research, particularly in developmental age [15]. It is relevant to note that the previous edition of the DSM reports “a body weight less than 85 percent of ideal body weight” as the required threshold for a diagnosis of “typical” AN (the only existing typology at that time) associated with evidence of amenorrhea, and thus provides an adaptable criterion to both developmental age and adult populations [8]. The DSM-5 removes this threshold, attributing a greater role to a clinician’s overall judgment in determining an ideal threshold [1]. A substantial number of recent studies, particularly on the developmental age group, agree that the threshold for diagnosis of AAN is a body weight equal to or greater than 85% of the average BMI for age [16,17,18]. Thresholds set at 90% of mean BMI by age [13] or the 10th percentile [19] have similarly been employed. Other studies have highlighted critical issues arising from the adoption of purely weight-based criteria to distinguish different mental disorders. Thomas et al. found that the moderate weight loss (about 1 kg) that frequently occurs on the first night of hospitalization, also due to changes in body fluids, led to a diagnostic change in favor of a picture compatible with AN “classic” awakening for 7.9% of a sample of patients admitted for other eating disorders [20]. These data and debates have led some researchers to propose the elimination of weight criteria for AN altogether while encouraging clinicians pay greater attention to each individual’s age, gender, weight trajectory, and bone and muscle mass to ensure that treatment remains as comprehensive as possible [21].

Focusing on FED psychopathology, many authors report that patients affected by AAN show more serious ED symptoms than patients with AN. Indeed, patients with AAN receive higher Eating Disorder Examination Questionnaire (EDE-Q) global scores [9]. A study also reports that adolescents with AN/AAN and premorbid overweight obtained greater ED severity scores on the EDE-Q Global scale and the EDE-Q Restraint, Weight Concerns, and Shape Concerns subscales, and displayed greater psychological morbidity on measures of anxiety and depression. These findings have been found independently of an ED diagnosis, which suggests that premorbid weight status may be a strong indicator of illness severity and psychological morbidity [22]. Since few studies focus on AAN, they also report inconsistent results as regards psychiatric comorbidities in this group of patients. On the one hand, the comparison between individuals diagnosed with AN and AAN shows that psychiatric comorbidity is almost twice as likely in individuals with typical AN [23]. Conversely, some studies indicate that adolescents with AAN exhibit higher rates of suicidality compared to their peers without eating disorders [3], yet show similar rates of self-harm and suicidality to those with AN. Additionally, research reveals that adolescents with AAN suffer from comparably poor self-esteem to those with AN [2]. Further studies report a high prevalence of obsessive–compulsive behaviors among adolescents with AAN, akin to what is observed in subthreshold conditions [24,25].

More generally, these considerations show the current difficulty in establishing diagnostic criteria that are uniquely valid for AAN while emphasizing the need for studies that specifically report on developmental trajectories and clinical phenotypes [6]. Previous research has documented that pediatric healthcare practitioners generally lack training in the assessment and management of restrictive eating disorders prevalent among individuals aged 10 to 14, such as AAN. These individuals may present in pediatric care settings, exhibiting acute medical instability and severe pathology associated with eating disorders. One significant challenge lies in the delayed identification of these cases by healthcare providers specializing in pediatrics, compounded by the reluctance of affected youth to engage in treatment [7]. Considering these issues, this review aims to compile data on AAN in the children and adolescents, in order to identify key epidemiological, psychopathological, organic, and treatment features of this condition.

## 2. Materials and Methods

This systematic review was conducted in May 2024. The review has been registered on the OSF (Open Science Framework) and is accessible at https://osf.io/fgq7u. It has also been assigned a specific DOI: https://doi.org/10.17605/OSF.IO/R7YQS (accessed on 1 May 2024).

The following databases were consulted: Medline (PubMed), Cochrane Library, and ClinicalTrials.gov. The search was broadened by searching the most relevant guidelines and clearinghouse websites, as well as the most important textbook sites. The Preferred Reporting Items for Systematic Reviews and Meta-Analyses (PRISMA) guidelines flowchart [26] was utilized to identify the clear exclusion of published material for specific reasons. Relevant selected works were summarized in terms of study design, participant number and kind, study type, and primary findings, as follows: inclusion criteria—published before November 2023; diagnostic criteria—individuals having a DSM-5-specified AAN; population—humans; language—English. Study designs included RCTs, cohort studies, cross-sectional studies, and retrospective investigations. Outcomes are recorded separately for persons with AAN. Articles on non-humans and in languages other than English were excluded. Study designs included descriptive studies, reviews, protocols, case reports, and case series. The outcomes were not reported. Search string: (atypical anorexia nervosa [MH]) AND (Child*, Adolescent*, Teen*, Young*, Youth*, Pediatr*, Infant). The initial search yielded 234 separate original articles.

Two independent investigators, FC and BV, examined the identified documents for appropriateness. A mediator, JP, arbitrated disputes between reviewers. At this stage, records were excluded:

- If released in a language other than English.
- If the study’s methodology—a narrative review, a systematic review, a study protocol, or any other study type—was unambiguously stated as such (RCT, cohort studies, cross-sectional studies, retrospective studies, etc. were included) or if the population that was included was unambiguously stated as being exclusively made up of animals other than humans.

The full texts of the records that remained after the initial screening were searched for retrieval. The obtained reports were then reviewed for eligibility by two independent reviewers. Records were excluded:

- If one of the exclusion criteria (population: non-humans, language: non-English, study design: descriptive studies, reviews, procedures, case report/case series) remained after suitability screening.
- If the study excluded children and adolescents or included a mixed group of children/adolescents and adults, but did not publish separate data for the two age groups.
- If no one was given a DSM-5-based diagnosis of AAN.

Two independent investigators (FC and BV) rated the technique of the selected works using JBI’s critical appraisal tools [27] for RCTs, cohort studies, and case–control studies, with differences among reviewers addressed through a mediator (JP). An overall evaluation (include/exclude/seek additional information) was determined. Records that passed both the screening and critical assessment processes were included in the final version of the review. A spreadsheet was used to categorize eligible studies for the systematic review. Due to limited available evidence, no studies were removed based on risk-of-bias scores.

Given the scarcity of randomized studies in this sector, we used the Newcastle–Ottawa Scale (NOS) quality assessment [28], which is a quality score for non-randomized research. This consists of two subsystems: a system for case–control studies that scores the three domains of “selection” (adequateness of the case definition, representativeness of the cases, selection of controls, definition of controls), “comparability” (comparability of cases and controls based on the design or analysis), and “exposure” (ascertainment of exposure, same method of ascertainment for cases and controls, non-response rate). A system for cohort studies was used for scoring the three domains of “selection” (representativeness of the exposed cohort, selection of the non-exposed cohort, ascertainment of exposure, demonstration that the outcome of interest was not present at the start of the study), “comparability” (comparability of cohorts based on the design or analysis), and “outcome” (assessment of outcome, follow-up long enough for outcomes to occur, adequacy of cohort follow-up) [28]. The collected findings were transformed to a quality scale (good/fair/poor) using a specific conversion system [29].

The presentation of the results has followed a framework aimed at providing data for clinical practice. Thus, four subsections were created: Diagnostic Criteria and Epidemiology, Eating Disorder-Specific and General Psychopathology, Organic and Neurobiological Findings, and Treatment Interventions.

## 3. Results

**Figure 1:**
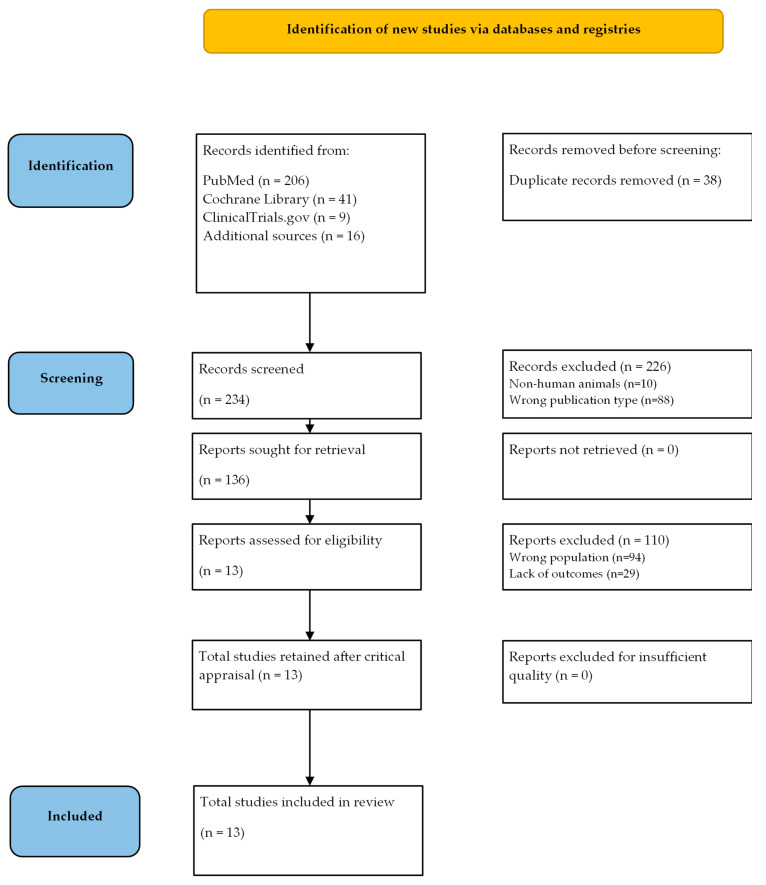
PRISMA diagram of the screening process.

Overall, 13 studies were retained in the final version of the review. The flowchart for the inclusion/exclusion process is given in Figure 1. The results of the included studies are systematically reported in Table 1. Ratings for the included papers are reported in Table 2.

### 3.1. Diagnostic Criteria and Epidemiology

Stice et al. investigated prevalence, incidence, duration, and course of DSM-5 eating disorders in a community sample of adolescent females aged over 8 years. By age 20, lifetime prevalence stood at 2.8 ± 1.5% for AAN. The same result (2.8 ± 1.5%) was documented for the cumulative incidence over an 8-year follow up. Incidence of AAN per 100,000 person-years was 366. The average episode duration reported was 11.6 (SD = 6.7, range = 1–22) months for AAN, while the remission rate at one year for this condition was 71% [21]. Forman et al. also provided relevant epidemiological data, conducting an analysis through the National Eating Disorders Quality Improvement Collaborative, focusing on patients with restrictive EDs, involving 700 adolescents aged 9–21 across 14 Adolescent Medicine FED programs. At program intake, 33.9% met the criteria for AAN. When comparted to those with AAN and AN, adolescents with ARFID tended to be male, younger, and had longer illness duration before seeking treatment [3].

Diagnostic persistence, crossover, and recovery at 9 or 18 months among individuals with multiple FED diagnoses, including AAN, was investigated by Breithaupt et al. The persistence over time of an AAN diagnosis was found less stable (AAN—restrictive 0.48; AAN—binging/purging 0.50) than ARFID (0.84). Crossover from binge eating/purging to restricting occurred 72% of the time, with the reverse happening 23% of the time. Stable recovery likelihood was limited in the study (0.00 to 0.36). Frequent diagnostic crossover between AN and AAN was documented, supporting continuity between typical and atypical presentations [32]. The theme of examined diagnostic changes from DSM-IV to DSM-5 criteria has been assessed by Fisher et al. The authors reported the percentages for DSM-IV diagnoses of AN (26.2%) and EDNOS (64.6%). All the patients with AN continued to present a diagnosis of AN, while the 198 patients previously diagnosed with EDNOS according to the DSM-IV were assigned different diagnoses in the DSM-5, except for 4 individuals who were classified as having an unspecified eating disorder. Among the DSM-5 diagnoses, 93 patients were identified as having AAN. In comparison to the patients diagnosed with AN, those exhibiting AAN were predominantly female (*p* < 0.05), slightly older (*p* < 0.05), and had significantly higher weight (99.2 vs. 83.0 mean% median BMI, *p* < 0.01). A trend indicating that non-white patients had a higher ratio of BN/purging disorder to AN/AAN compared with white patients was reported [34].

### 3.2. Eating Disorder-Specific and General Psychopathology

Specific data on psychopathology were identified. When considering the previously cited sample enrolled by Garber et al. (adolescents and young adults diagnosed with AN and AAN), individuals with AAN demonstrated higher Eating Disorder Examination Questionnaire (EDE-Q) global scores compared to those with AN [9].

In another study, an analysis of the initial presentations of adolescents with AAN (n = 42) and full-threshold AN (n = 118) at a specialized pediatric eating disorder program, both groups exhibited similar rates of psychiatric comorbidities (38% vs. 45%) and suicidal ideation (43% vs. 39%). However, distress linked to eating and body image was more severe in adolescents with AAN [2]. In the research by Stice et al., for participants with AAN, greater functional impairment, emotional distress, and suicidality were documented when compared to non-eating-disordered participants [3]. When compared to healthy controls (HCs) in the study of Olivo et al. on adolescent females with AAN, significant differences emerged between patients and HCs in BMI (*p* < 0.002), EDE-Q total score (*p* < 0.000), and Obsessive–Compulsive Inventory (OCI-R) total score (*p* < 0.000) [35]. As documented in other research covering psychopathological evaluation, individuals with AAN showed higher ED psychopathology than those with AN-R and ARFID (*p* < 0.001). Specifically, the Drive for Thinness, Bulimia, and Body Dissatisfaction scales exhibited higher levels within the AAN group [4]. These results were expanded by Pauls et al., showing that compared to those with AN-R, AAN adolescents exhibited more severe drive for thinness (*p* = 0.011) and higher body dissatisfaction (*p* = 0.038), but better global functioning (*p* = 0.032). Those with high adverse childhood experiences (ACEs) questionnaire scores (ACE score ≥ 4) were over five times as likely to have AAN (*p* = 0.008) [37].

The reviewed studies provide nuanced insights into the psychopathological profiles of adolescents with AAN. Garber et al. (good quality) found higher Eating Disorder Examination Questionnaire (EDE-Q) global scores in AAN compared to AN subjects. At a specialized pediatric eating disorder program (poor quality), adolescents with AAN and full-threshold AN showed similar psychiatric comorbidity rates, but more severe distress related to eating and body image in those with AAN. Stice et al. (good quality) reported greater functional impairment, emotional distress, and suicidality in AAN compared to non-eating-disordered individuals. Olivo et al. (good quality) observed significant differences in BMI, EDE-Q scores, and Obsessive–Compulsive Inventory (OCI-R) scores between AAN adolescents and healthy controls. Psychopathological evaluations (fair quality) highlighted higher eating disorder symptomatology in AAN subjects, particularly in Drive for Thinness, Bulimia, and Body Dissatisfaction scales, compared to those with AN-R and ARFID. Pauls et al. (fair quality) noted more severe drive for thinness and body dissatisfaction, but better global functioning in AAN compared to AN-R subjects, with higher ACE scores associated with AAN. These findings underscore the substantial psychopathological burden in AAN, with caution needed in interpreting lower-quality study results due to potential methodological limitations.

### 3.3. Organic and Neurobiological Findings

Disease-specific organic features have been documented for children and adolescents with AAN. Lower heart rate has been associated with a faster rate of weight loss, while lower serum phosphorus levels were linked to greater and longer-duration weight loss, independent of admission weight [9]. The assessments performed by Sawyer et al. documented specific results for AAN as well. Their findings demonstrated that compared to AN, a higher percentage of adolescents with AAN were previously overweight or obese (71% vs. 12%). Those with AAN experienced greater weight loss (17.6 kg vs. 11.0 kg) over a longer duration (13.3 vs. 10.2 months). Notably, there were no significant differences in the frequency of bradycardia (24% vs. 33%) or orthostatic instability (43% vs. 38%). In other research, individuals with ARFID typically showed a BMI lower than 81.1% of their counterparts, while those with AAN exhibited an average BMI comparable to 48% of their peers [2].

As identified by Kimber et al., exploring practitioners’ perspectives on differences between adolescents with AAN versus AN, those with AAN tend to have higher pre-existing weight and encounter more weight-based teasing compared to their AN counterpart. Specific clinical challenges identified by practitioners in working with AAN included navigating conflicting messages about eating disorders and weight loss, understanding a justified fear of weight gain, and addressing heightened risks of parental and therapist collusion with the eating disorder [30]. Relevant data on premorbid weight have been confirmed and expanded by Matthews et al., showing a 29.6% rate of premorbid overweight/obesity among 253 hospitalized patients (aged 10–22) with AN/AAN. Those with AN/AAN and premorbid overweight/obesity were more frequently cisgender male (24% vs. 8.4%), diagnosed with AAN (62.7% vs. 32%), and had lost a greater percentage of body weight (29% vs. 16.4%) compared to premorbid normal-weight counterparts [22].

In the previously cited study by Forman et al., the authors found a positive change in mean percentage median body mass index (percentage median BMI—%MBMI) across all sites at 1-year follow-up. %MBMI at intake emerged as a significant predictor of weight recovery, showing a 12.7% change in the model [33]. In the previously cited first study by Olivo et al. investigating brain structure using voxel-based morphometry in AAN and HCs, no discernible differences in gray matter regional volume between the groups were detected [35]. The same group explored the neural circuitry associated with the hedonic response between AAN and HC. Participants viewed images of high- and low-calorie food in alternating blocks, analyzing the high-calorie > low-calorie contrast. AAN exhibited increased connectivity in pathways linked to multimodal somatosensory processing and memory retrieval. However, connectivity decreased in salience, attentional networks, and cerebello-occipital network in patients. Notably, AAN patients showed heightened somatosensory processing in response to high-calorie food images compared to controls, yet high-calorie food failed to evoke robust salient bottom-up responses in patients. Additionally, no increased connectivity was observed in inhibitory control regions. These findings suggest distinct psychopathological mechanisms underlying food restriction in AAN compared to AN [36].

### 3.4. Treatment Interventions

No disease-specific structured protocol to treat children and adolescents with AAN emerged from the present review. Nonetheless, Gledhill et al. evaluated a novel training program’s effectiveness in altering participants’ perceptions of body size. Through a unique adaptation of a cognitive bias training regimen, participants engaged in body size judgments of female figures across four daily training sessions, aiming to enhance accuracy. Study 1 included young women with high body size concerns in a randomized controlled trial, showcasing significant improvement in body size judgments and reduced eating concerns post-training. Study 2 applied the program to women with AAN, demonstrating positive shifts in body size perception and reduced eating-disordered concerns [31].

## 4. Discussion

This review aims to systematically synthesize the diagnostic nuances and clinical intricacies characterizing AAN among children and adolescents. By amalgamating diverse research findings, this exploration aims to illuminate the epidemiological, psychopathological, and organic dimensions of AAN, offering an understanding of this distinct subset within FED. AAN among children and adolescents presents a complex landscape encompassing nuanced diagnostic criteria, psychological intricacies, organic correlations, and treatment interventions. Exploring the extensive research reveals multifaceted aspects defining AAN across various dimensions.

The available evidence provides a detailed understanding of diagnosis and epidemiology of AAN in adolescence. Stice et al.’s [3] high-quality data indicate that the lifetime prevalence and cumulative incidence by age 20 are both 2.8%, with an average episode duration of 11.6 months. Their study also shows a one-year remission rate of 71%. Forman et al. [33] reveal that 33.9% of adolescents in their study met the criteria for AAN, with significant differences in demographic characteristics compared to those with AN and ARFID. However, Breithaupt et al.’s [32] lower-quality study highlights diagnostic instability and frequent crossover between AAN subtypes, indicating the need for more robust research to better understand these dynamics.

The reviewed studies provide nuanced insights into the psychopathological profiles of adolescents with AAN. Garber et al. (good quality) found higher EDE-Q global scores in AAN compared to AN [9]. At a specialized pediatric eating disorder program (poor quality), adolescents with AAN and full-threshold AN showed similar psychiatric comorbidity rates, but more severe distress related to eating and body image in AAN [2]. Stice et al. (good quality) reported greater functional impairment, emotional distress, and suicidality in AAN compared to non-eating-disordered individuals [3]. Olivo et al. (good quality) observed significant differences in BMI, EDE-Q scores, and obsessive–compulsive scores between AAN adolescents and healthy controls [35]. Psychopathological evaluations (fair quality) highlighted higher eating disorder symptomatology in AAN, particularly in drive for thinness, bulimia, and body dissatisfaction, compared to AN-R and ARFID [4]. Pauls et al. (fair quality) noted more severe drive for thinness and body dissatisfaction, but better global functioning in AAN compared to AN-R, with higher ACE scores associated with AAN [37]. Incorporating insights from cross-cultural psychology, recent research underscores significant cultural influences on cognitive mechanisms involved in emotion perception, particularly in interpreting facial expressions. Findings reveal that cultural norms, such as display rules and cognitive styles, shape how individuals from different backgrounds perceive and categorize emotional intensity. For instance, studies indicate distinct patterns in emotion perception between US, Japanese, and Russian participants, highlighting variations in attentional biases and the categorization of emotion prototypes across cultures. These cultural nuances underscore the need for further exploration into how cross-cultural differences may influence cognitive and emotional aspects relevant to eating disorders like AN, suggesting a critical area for future research and clinical consideration [38].

These findings underscore the substantial psychopathological burden in AAN, with caution needed in interpreting lower-quality study results due to potential methodological limitations.

Organic and neurobiological findings in adolescents with AAN reveal distinctive characteristics compared to traditional AN. Garber et al. (good quality) associate lower heart rate with faster weight loss and lower serum phosphorus levels with prolonged weight loss in AAN [9]. Sawyer et al. (good quality) note that adolescents with AAN often have a history of overweight or obesity and experience more significant and prolonged weight loss compared to AN counterparts. However, frequencies of bradycardia and orthostatic instability were similar between AAN and AN [2]. Kimber et al. (good quality) highlight that adolescents with AAN frequently encounter weight-based teasing and pre-existing weight issues [30]. Matthews et al. (fair quality) report a higher prevalence of overweight/obesity among hospitalized patients with AAN compared to those with normal weight, with associated gender differences and more substantial weight loss [22]. Regarding neurobiological aspects, Olivo et al. (good quality) found no structural brain differences using voxel-based morphometry, while functional connectivity studies (Olivo et al., fair quality) suggest altered neural circuitry in AAN related to food stimuli, indicating distinct mechanisms from AN [35,36]. These findings underscore the need for further research to refine understanding and inform tailored treatment strategies for AAN.

From the present review, no structured disease-specific protocol for treating children and adolescents with AAN has emerged. However, Gledhill et al. explored the effectiveness of a novel training program designed to alter participants’ perceptions of body size. This program adapted cognitive bias training, involving participants in daily sessions where they judged female figures’ body sizes to improve accuracy. In Study 1, which included young women with high body size concerns in a randomized controlled trial, significant improvements were noted in body size judgments and reduced eating concerns after the training. Study 2 extended this program to women specifically diagnosed with AAN, demonstrating positive shifts in body size perception and reduced eating-disordered concerns [31]. These findings suggest promising avenues for intervention in AAN, emphasizing the potential benefits of tailored cognitive interventions in addressing body image and eating disorder concerns.

### Strengths and Limitations

Strengths of this review lie in the comprehensive integration of diverse studies exploring various facets of AAN in children and adolescents. The inclusion of clinical trials, neurobiological investigations, and psychosocial analyses offers a multifaceted understanding of the disorder, providing a nuanced view of its organic, psychological, and behavioral dimensions. The review’s strength also resides in synthesizing findings from multiple studies, showcasing a comprehensive picture of the distinctive features characterizing AAN compared to traditional AN. Additionally, the review highlights gaps in current research, pinpointing areas requiring further exploration, thereby guiding future investigative endeavors. However, certain limitations should be acknowledged. The variability in methodologies and sample sizes across the studies reviewed might introduce inconsistencies or biases in the synthesized conclusions. The predominantly cross-sectional nature of many studies limits the establishment of causal relationships or the ability to track the longitudinal trajectory of AAN. Moreover, the scarcity of studies specifically focusing on AAN might constrain the depth of understanding of this variant compared to more extensively studied eating disorders. Furthermore, the potential for publication bias or selective reporting across studies could affect the comprehensiveness of the review’s conclusions. Despite these limitations, this review provides a valuable synthesis of current knowledge, setting a foundation for future research directions in the domain of AAN among children and adolescents.

## 5. Conclusions

In conclusion, this exploration of AAN in children and adolescents unveils critical insights into the distinctive nature of this eating disorder variant. The synthesis of evidence from diverse studies underscores the significance of weight history in assessing illness severity and progression. AAN manifests with pronounced psychopathological profiles, as evidenced by elevated distress related to eating and body image, particularly when compared to traditional AN. Noteworthy findings emphasize the need for tailored interventions acknowledging the challenges faced by individuals with AAN, notably those related to weight-based teasing, conflicting perceptions of eating disorders, and fear of weight gain. Despite similarities in severity to AN, AAN exhibits nuanced differences, demanding a deeper understanding of its psychosocial experiences and clinical presentations. This review highlights the dearth of research specifically dedicated to AAN, signaling a critical need for more comprehensive and longitudinal investigations. Moving forward, prioritizing longitudinal studies, standardized methodologies, and interventions addressing psychosocial distress and childhood adversity will be pivotal in advancing our understanding and treatment approaches for this distinctive variant of eating disorders.

## Figures and Tables

**Table 1:** Summary of the collected evidence.

Study	Patients	Intervention (Administered Diagnostic Tool/Clinical Intervention)	Comparison	Outcome
[9]	AAN = 50, AN = 66; age: 16.5 ± 2.6 years; F = 91%	Proctored questionnaires, growth records, medical history, socioeconomic status, date of last menstruation; biochemicals and instrumental analysis; self-administered EDE-Q	AN	Historical BMI, %mBMI and %mBMI at admission higher in AAN. No significant differences in weight history, amenorrhea, lowest heart rate and electrolytes. Higher systolic blood pressure, EDE-Q and all subscales except “concern for nutrition” higher in AAN.
[2]	AAN = 42, AN = 118; age: 15.49 vs. 15.4 years; F = 88% (both groups)	Recording of anthropometric data, eating disorder symptoms and psychiatric comorbidities	AN	In AAN: higher rates of premorbid overweight or obesity, weight loss over longer periods, related discomfort to nutrition and body size
[3]	496 females completing annual diagnostic interviews over 8 years; 12–15 years	BMI, ED symptoms, Social Adjustment Scale-Self Report for Youth, K-SADS, frequency of visits to mental health	AN, BN, BED, subthreshold BN, subthreshold BED, PD, EDNOS and controls	AAN episodes last longer than other EDs, and AAN showed higher psychopathological distress, excluding suicidal risk in AN and BMI in BN, subthreshold BN, and BED.
[30]	Comments of 23 practitioners on AN and AAN adolescents	Individual semi-structured interview administered to 23 professionals of health from four countries	AN	AAN often starts at higher pre-existing weights and receives more weight-based teasing. Unique hurdles, such as navigating mixed messages about eating disorders and weight loss, understanding and supporting the legitimate fear of gaining weight, and addressing the heightened risks of collusion.
[31]	Study 1: 40 students (1–20: 18.2 ± 0.4 years, 21–40: 19 ± 1.3 years), F. Study 2: 21 AAN; F = 100%; age: 27.7 ± 7.5 years	Training program to recalibrate perception of body size Study 1: BMI; BSQ, EDE-Q, BDI, RSE; categorical perception task with computer-generated images. Study 2: BMI, BSQ, EDE-Q; WAIS-R IQ Digit Span task	Study 1: college students without FED; Study 2: only F with AAN	Study 2: In AAN, full training is associated with a cumulative shift in the categorical boundary toward heavier bodies and significant reductions in the EDE-Q subscales related to food restriction and body size concerns persisted for up to one month
[32]	AAN = 26; AN = 40; ARFID = 16; age: 10–23 years	Semi-structured interviews	AN and ARFID	Persistence of binge eating/purging and atypical presentations (AAN) showed less consistency over follow-up. Notable transitioning between binge eating/purging and restricting behaviors.
[33]	AAN = 238, AN = 375, ARFID = 87; F = 83.2% vs. 90.4% vs. 77.0%; age: 9–21 years	Demographic data, weight and height at intake and follow-up, treatment before intake and treatment during the follow-up year	AN and ARFID	AAN: fewer males than ARFID; ARFID were younger, had longer illness duration before presentation, and fewer prior visits compared to AAN. Significant differences in %MBMI at intake and regular menses, with AAN showing distinct patterns. Higher %MBMI at intake predicted weight recovery significantly. AAN had higher odds of weight recovery compared to ARFID.
[34]	AN = 100, AAN = 93, ARFID = 60, BN = 29, PD = 18, UFED = 4, subthreshold BN = 2, subthreshold BED = 2, BED = 1.	Gender, age, ethnicity, weight, height, diagnosis according to DSM-IV and DSM-5	AN, ARFID, BN, PD, UFED, subthreshold BN, subthreshold BED, BED	AAN more frequent females, slightly older age, and higher weights than AN. ARFID: higher male representation and younger age than AAN. Non-white patients trend toward higher ratio of BN/PD to AN/AAN compared to white patients.
[35]	AAN = 22; HC = 38; F = 100%; age: 14.7 vs. 14.8 years.	Basic clinical data; EDE-Q, BIS-11, nd OCI-R; study of brain structure through voxel-based morphometric analysis of MRI	HCs	Significant differences in terms of BMI, EDE-Q total score and OCI-R; no differences in regional gray matter volume could be detected
[36]	AAN = 28; HC = 33; F = 100%; age: 14.8 ± 1.6 vs. 15.3 ± 1.3 years.	Basic clinical data, semi-structured interviews. Clinical intervention: FBT and periodic follow-up; images of high- or low-calorie foods were shown in alternating blocks during functional MRI, contrast was calculated	HCs	When comparing images of high- and low-calorie foods, AAN subjects demonstrated greater connectivity than HCs: enhanced connections from the right anterior superior temporal gyrus, and decreased connectivity in the left area.
[4]	AAN = 59, AN-R = 193, BED = 14, ARFID = 94; F = 83%; age 15.8 ± 2.5 years	EDI-3, CDI, MASC, CBCL	AN and ARFID	AAN: higher EDI-3 (drive for thinness, bulimia, body dissatisfaction) scores (AAN > AN > ARFID); CDI higher in AAN and AN than in ARFID; no differences in terms of interpersonal insecurity, interoceptive deficit, emotional dysregulation and maturity fears.
[37]	AAN = 42, AN-R = 79; F = 88.1% vs. 93.7%; age: 15.9 vs. 15.6 years;	Examination of psychological morbidity and exposure to adverse childhood experiences exploring the connection between reaching the target weight and the likelihood of rehospitalization in adolescents with AN and AAN.	AN-R	AAN subjects had more severe drive for thinness, body dissatisfaction, and lower quality of life, but better global functioning. Adolescents with high adverse childhood experiences had over 5 times the odds of having AAN. No difference on low self-esteem or self-injury
[22]	Overall, 253 patients with AN or AAN; age: 10–22 years; 29.6% with and 70.4% without premorbid overweight or obesity	Retrospective review of clinical (sex) and psychopathological data, related to the ED	AN	Patients with/without premorbid overweight or obesity: more often cisgender males, diagnosed with AAN, and had lost a greater percentage of body weight.

Abbreviations: AAN: atypical anorexia nervosa; AN: anorexia nervosa; AN-R: anorexia nervosa, restrictive subtype; ARFID: avoidant/restrictive food intake disorder; BED: binge-eating disorder; BN: bulimia nervosa; BSQ: body size concern; CBCL: Child Behavior Checklist; CDI: Children’s Depression Inventory; ED: eating disorder; EDE-Q: Eating Disorder Examination Questionnaire; EDI: Eating Disorder Inventory; HC: healthy control; K-SADS: Kiddie Schedule for Affective Disorders and Schizophrenia; MRI: magnetic resonance imaging; UFED: unspecified feeding and eating disorder.

**Table 2:** Quality assessment of the included studies (case reports excluded).

Study	Selection	Comparability	Exposure (Case–Control)/ Outcome (Cohort)	Quality	Notes
[9]	**	**	***	Good	
[2]	**		**	Poor	
[3]	***	*	***	Good	
[30]	/	/	/	/	Qualitative study
[31] + Study 2	***	**	***	Good	
[32]	***	**	***	Good	
[33]	***	**	***	Good	
[34]	**		*	Poor	
[35]	***	**	***	Good	
[36]	***	**	***	Good	
[4]	**	**	**	Fair	
[37]	**	*	**	Fair	
[22]	**	**	**	Fair	

+ Newcastle–Ottawa Scale for cohort studies used to assess this paper. The number of asterisks (*, **, ***) indicates the overall quality scoring according to Newcastle–Ottawa Scale.
